# New Appliances and Things Medical

**Published:** 1903-11-21

**Authors:** 


### NEW APPLIANCES AND THINGS MEDICAL.

### NICHOLSON'S S. S. WHISKY.

(J. & W. Nicholson & Co., Limited, Distillers and Rectifiers, London).

Our examination of this blend of Scotch whiskies convinces us that it is old and well matured, which fact is of especial importance when it is to be medicinally administered. The flavour is soft and mellow and its acidity but slight. Both for therapeutic purposes and general use this wholesome blend should be useful and reliable.

### PLASMON PREPARATIONS.

(International Plasmon, Limited, 66a Farringdon Street, London, E.C.).

In practical dietetics the want of a tasteless, compact, easily digested and cheap preparation of pure proteid has often been felt. In Plasmon we have such a preparation, which consists of the proteids of milk rendered soluble by combination with bicarbonate of soda. It is a yellowish-white powder, easily soluble in warm fluids and devoid of taste. Its nutritive value is very high as it consists of 90 per cent. proteid, and one may roughly state that one part of plasmon is equal as a source of proteid to four parts of meat, while its tastelessness and solubility enables it to be added to other foods, greatly raising their nutritive value. Plasmon Arrowroot is prepared from the finest arrowroot with a suitable proportion of plasmon added, which constitutes a highly nourishing food most valuable to invalids. Plasmon Chocolate, containing 25 per cent. of plasmon, forms an admirable concentrated form of highly palatable nutriment, and should be of immense value as an emergency ration. Plasmon Biscuits are also made in many varieties by Messrs. Peek, Frean and Co., containing 20 per cent. of plasmon. These preparations mark an important advance in dietetics which we heartily welcome.

### ARABELLA.

(Arabella, Limited, 2a Hay Hill, Berkeley Square, London, W.).

This natural mineral water from Austria-Hungarian springs contains, according to the published analysis, a large proportion of magnesium and sodium sulphate in solution. From its composition one would expect this medicinal water to be far less palatable and it is certainly surprising to find so little unpleasantness in this respect. As a gentle laxative or in various hepatic derangements "Arabella" will probably gain the same reputation in this country as it already possesses on the continent.

